# A Novel Adaptive Modulation Based on Nondata-Aided Error Vector Magnitude in Non-Line-Of-Sight Condition of Wireless Sensor Network

**DOI:** 10.3390/s18010229

**Published:** 2018-01-15

**Authors:** Fan Yang, Xiaoping Zeng, Haiwei Mao, Xin Jian, Xiaoheng Tan, Derong Du

**Affiliations:** 1 College of Communication Engineering, Chongqing University, Chongqing 400044, China; Stevenyang9016@cqu.edu.cn (F.Y.); 20161213067@cqu.edu.cn (H.M.); jianxin@cqu.edu.cn (X.J.); txh@cqu.edu.cn (X.T.); dr_du@cqu.edu.cn (D.D.); 2 Chongqing Jinmei Communication Co. Ltd., Chongqing 400030, China

**Keywords:** adaptive modulation, nondata-aided error vector magnitude, non-line-of-sight, *η*−*μ* fading channel, wireless sensor network

## Abstract

The high demand for multimedia applications in environmental monitoring, invasion detection, and disaster aid has led to the rise of wireless sensor network (WSN). With the increase of reliability and diversity of information streams, the higher requirements on throughput and quality of service (QoS) have been put forward in data transmission between two sensor nodes. However, lower spectral efficiency becomes a bottleneck in non-line-of-sight (NLOS) transmission of WSN. This paper proposes a novel nondata-aided error vector magnitude based adaptive modulation (NDA-EVM-AM) to solve the problem. NDA-EVM is considered as a new metric to evaluate the quality of NLOS link for adaptive modulation in WSN. By modeling the NLOS scenario as the η−μ fading channel, a closed-form expression for the NDA-EVM of multilevel quadrature amplitude modulation (MQAM) signals over the η−μ fading channel is derived, and the relationship between SER and NDA-EVM is also formulated. Based on these results, NDA-EVM state machine is designed for adaptation strategy. The algorithmic complexity of NDA-EVM-AM is analyzed and the outage capacity of NDA-EVM-AM in an NLOS scenario is also given. The performances of NDA-EVM-AM are compared by simulation, and the results show that NDA-EVM-AM is an effective technique to be used in the NLOS scenarios of WSN. This technique can accurately reflect the channel variations and efficiently adjust modulation order to better match the channel conditions, hence, obtaining better performance in average spectral efficiency.

## 1. Introduction

A wireless sensor network (WSN) as a wireless network is widely applied in environmental monitoring, invasion detection, disaster aid and various other fields [1,2,3]. Higher requirements on throughput and quality of service (QoS) are brought forward in data transmission between two sensor nodes based on the reliability and diversity of information streams [2,4,5]. However, the problem of lower spectral efficiency in non-line-of-sight (NLOS) condition becomes a bottleneck [6], which restricts the high data rate communications in WSN. Adaptive modulation enables robust and spectrally-efficient transmission [7], which allows the system to adapt the modulation scheme according to the channel conditions in order to improve network average spectral efficiency [8]. The basic premise of adaptive modulation in NLOS scenario is to estimate the channel quality at the receive sensor node and feed it back to the transmitted sensor node, so that the transmission scheme can be adapted relative to the channel characteristics. Obviously, the key to adaptive modulation in NLOS link focuses on which metric is fit for, and how to make the adaptation strategy based on the metric.

Signal-to-noise ratio (SNR) as a traditional performance metric has been used in evaluation channel quality [9,10,11]. Many existing works that approach the adaptive modulation problem are based on DA-SNR. In [12], the authors suggested preamble-aided channel quality estimation (i.e., DA-SNR) as a metric for channel adaptation to improve the link quality under dynamic interference of WSN. In [13], the authors explored the scheme by evaluation DA-SNR to select the modulation order for the transmitter; and Hanzo and Torabi et al. [14,15] optimized the switching levels of DA-SNR to improve the throughput performance. However, DA-SNR-AM research mostly focuses on DA-SNR estimation and switching level optimization for certain special channels, such as additive white Gaussian noise (AWGN) and Rayleigh fading channels, which cannot provide the general model for NLOS propagation scenario in a realistic WSN. On the other hand, the fixed time interval between these known data sequences results in a non-real-time evaluation of channel quality estimation, especially in the case of rapidly time-varying channels. Recently, the η−μ distribution as a general fading distribution fits for the NLOS scenario [16], and it is proposed for modeling the fading channel in NLOS condition of WSN [17]. Meanwhile, the nondata-aided error vector magnitude (NDA-EVM) as a new performance metric is becoming increasingly popular in quantifying the performance of communication system [18].

This paper proposes NDA-EVM as a new performance metric for adaptive modulation (i.e., NDA-EVM-AM) in an NLOS scenario. The NDA-EVM reflects the dispersion range of the received symbols from the ideal constellation points [18]. As a symbol-level performance metric, it is sensitive to the degradation of a wireless system [19], and fluctuations in the NDA-EVM can reveal even small variations in channel fading. Unlike DA-SNR, the NDA-EVM can be used to infer some achievable system performance (e.g., the symbol error rate (SER), throughput and outage probability) even if packet reception fails [20]. Hence, characterizing the channel quality using NDA-EVM is preferred. However, there is a dearth of studies applying NDA-EVM to adaptive modulation in the NLOS scenario. This can be attributed to two reasons. First, the current literature does not provide closed-form expression of the NDA-EVM in an NLOS scenario. Hence, modeling the channel of NLOS scenario by using the η−μ distribution and providing the closed-form expression of the NDA-EVM under the η−μ fading channel are the theoretic basis of NDA-EVM-AM. Second, due to the lack of the expression for the relationship between NDA-EVM and SER, the adaptation strategy based on NDA-EVM remains an open problem. Thus, providing the closed-form expression of SER-NDA-EVM is the key for the design of the adaptation strategy. 

Against this background, the primary contributions of this paper can be summarized as follows. (1) A closed-form expression is derived for the NDA-EVM of the multilevel quadrature amplitude modulation (MQAM) signal over η−μ fading channels, and the relationship between NDA-EVM and SER is also formulated; (2) A NDA-EVM state machine is designed for adaptation strategy of NLOS scenario in WSN; (3) The theoretical outage capacity of NDA-EVM-AM in NLOS scenario is given.

The paper is structured as follows. In Section 2, the system model of NDA-EVM-AM in NLOS scenario is presented. In Section 3, NDA-EVM as a channel metric in the NLOS scenario is formulated, the closed-form expression of SER in terms of NDA-EVM is also presented. In Section 4, the adaptation strategy based on NDA-EVM state machine is designed. In Section 5, the algorithmic complexity of NDA-EVM-AM is analyzed. In Section 6, the theoretical outage capacity of NDA-EVM-AM in NLOS scenario is presented, while, in Section 7, the numerical results obtained by simulations are shown by comparing the performance of NDA-EVM-AM. Finally, the conclusions are drawn in Section 8. 

## 2. System Model of NDA-EVM-AM in an NLOS Scenario

For the case of a single-input single-output (SISO) system, we consider two sensor nodes adaptively adjust modulation order to transmit the information in an NLOS scenario of WSN. Since a variable-power scheme increases the peak level of co-channel interference [21] imposed on other sensor nodes of WSN, the transmitter keeps constant-power in adaptive modulation. The information sequence d[n] is mapped into complex MQAM signals of modulation order M (e.g., x[n]); then, M∈{4,16,64,256}. Considering the η−μ fading channel as a channel model in NLOS condition, MQAM signals received can be written as:

(1) $$y[n]=ge^{-j\theta }x[n]+w$$

where w is an independent and identically distributed (i.i.d) complex Gaussian noise with a zero mean and variance $\sigma_{n}^{2}$; and g and θ are the channel gain and phase shift, respectively, which are both introduced by the fading channel. To achieve the best performance, the optimum receiver for the signal received is consists of matched filter, whose output is multiplied by the corresponding complex-valued (conjugate) channel gain $ge^{j\theta }$. The effect of this multiplication is to compensate for the phase shift in the channel. Assuming after matched filtering and sampling with perfect symbol timing, the phase shift θ can be compensated without error, the baseband discrete-time complex-valued signal is obtained as [22,23]:

(2) y[n]=αx[n]+w

In Equation (2), α is attenuation factor (i.e., 0<α<1), which can be obtained by normalize channel gain. Due to the probability density function (PDF) of the fading power $z=\alpha^{2}$ can be expressed as [24,25,26,27],

(3) $$f_{Z,\eta -\mu }(z)=\frac{z^{2\mu -1}}{\theta_{1}^{s}\theta_{2}^{s}\Gamma (2\mu )}\Phi_{2}^{\left(2\right)}(\mu ,\mu ;2\mu ;\frac{-z}{\theta_{1}},\frac{-z}{\theta_{2}})$$

where $\Phi_{2}^{\left(2\right)}(\cdot )$ is the confluent Lauricella function [28], $\theta_{1}=\frac{1}{2\mu (h+H)}$ and $\theta_{2}=\frac{1}{2\mu (h-H)}$, where $h=\frac{2+\eta^{-1}+\eta }{4}$, $H=\frac{\eta^{-1}-\eta }{4}$ in Format 1 and $h=\frac{1}{1-\eta^{2}}$, $H=\frac{\eta }{1-\eta^{2}}$ in Format 2; and $\mu =\frac{E^{2}\{\alpha^{2}\}}{2\mathrm{var}\{\alpha^{2}\}}[1+{\left(\frac{H}{h}\right)}^{2}]$. The PDF of α can be derived as

(4) $$f_{\alpha ,\eta -\mu }(\alpha )=\frac{2\alpha^{4\mu -1}}{\theta_{1}^{\mu }\theta_{2}^{\mu }\Gamma (2\mu )}\Phi_{2}^{\left(2\right)}(\mu ,\mu ;2\mu ;\frac{-\alpha^{2}}{\theta_{1}},\frac{-\alpha^{2}}{\theta_{2}})$$

**Figure 1:**
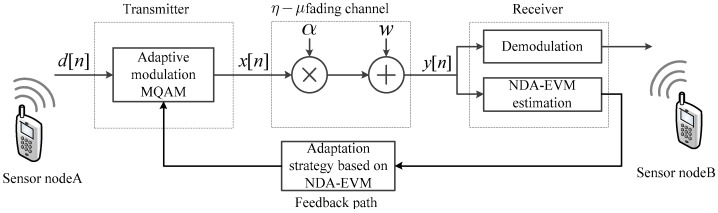
The system model of nondate aided error vector magnitude based adaptive modulation (NDA-EVM-AM) between the sensor nodes.

The system model of adaptive transmission between two sensor nodes is illustrated in Figure 1. We estimate the NDA-EVM at time n and adapt the modulation order, the adaptation takes place at a multiple of the symbol duration. After MQAM signals are transmitted through η−μ channels, the receiver estimates the NDA-EVM to evaluate the quality of the channel according to the received MQAM signals. The availability of this channel quality metric at the transmitter allows it to adapt its transmission scheme relative to the channel variation. The adaptation strategy is modeled as NDA-EVM state machine where each state is represented by a couple formed by a modulation order. This issue will be discussed in more detail in Section 4. We neglect the delay of feedback between two sensor nodes and assume that the feedback path does not introduce any errors.

## 3. Channel Quality Metric in WSN

NDA-EVM is defined as the root-mean-squared (RMS) value of the received symbols y and the estimated transmitted symbols $\hat{x}$, the expression can be expressed as

(5) $$\xi [M]=\sqrt{\sum_{n=1}^{N}{\left|y[n]-\hat{x}[n]\right|}^{2}/NP_{0}}$$

where y[n] is the nth received symbols and $\hat{x}[n]$ can be obtained from the received symbols by using the ML estimation. The symbol power is normalized, $P_{0}=1$. For simplicity, the index n is dropped for the remainder of this discussion. Considering x is a MQAM signals with modulation order M, then $x\in \{S_{1},\ldots ,S_{i},\ldots ,S_{M}\}$, $S_{i}$ is the ith constellation point in the constellation set, which can be expressed as

(6) $$\begin{array}{l} S_{i}=S_{i,R}+jS_{i,I}=(2i-k)b\;+\;j(2m-k)b,\\ i,m=0,1,\cdot \cdot \cdot k;b\;=\sqrt{3/2(M-1)}\;\&\;k=\sqrt{M}-1\; \end{array}$$

where b is the normalized symbol amplitude. Because of the independence and symmetry of the real and imaginary parts of the MQAM symbols, only the real part of the signal is considered. Thus, we can rewrite Equation (5) as

(7) $$\begin{array}{l} \xi [k{]}^{2}=2E\{(y_{R}-{\hat{x}}_{R}{)}^{2}\}\\ =2\sum_{i=0}^{k}P({\hat{x}}_{R}=S_{i,R})\int_{-\infty }^{+\infty }(y_{R}-S_{i,R}{)}^{2}f(y_{R}|{\hat{x}}_{R}=S_{i,R})dy_{R} \end{array}.$$

By using Equation (2), the conditional PDF of the received symbol $y_{R}$ is

(8) $$f(y_{R}|x_{R}=S_{i,R})=\frac{1}{\sigma_{n}}\varphi (\frac{y_{R}-\alpha S_{i,R}}{\sigma_{n}}),$$

where φ(⋅) is the PDF of a standard normal distribution. By the total probability theorem:

(9) $$P({\hat{x}}_{R}=S_{i,R})=\sum_{j=0}^{k}P(x_{R}=S_{j,R})\int_{D_{i,R}}f(y_{R}|x_{R}=S_{j,R})dy_{R}.$$

where $D_{i,R}$ is the decision region for symbol $S_{i,R}$, which is confirmed by the ML Criterion,

(10) $$D_{i,R}=\{\begin{array}{ccc} -\infty <y_{R}\leq S_{0,R}+b, & if & i=0;\\ S_{i,R}-b<y_{R}\leq S_{i,R}+b & if & 1\leq i\leq k-1;\\ S_{k,R}-b<y_{R}<\infty & if & i=k. \end{array}$$

All constellation points are transmitted with equal probability such that $P(x_{R}=S_{j,R})=1/(1+k),\forall j$. From the definition of conditional probability, $f(y_{R}|{\hat{x}}_{R}=S_{i,R})$ is evaluated as follow

(11) $$f(y_{R}|{\hat{x}}_{R}=S_{i,R})=\frac{\sum_{j=0}^{k}f(y_{R}|x_{R}=S_{j,R})}{\sum_{j=0}^{k}\int_{D_{iR}}f(y_{R}|x_{R}=S_{j,R})dy_{R}},\;y_{R}\in D_{i,R}$$

Using Equations (9) and (11), the expression in Equation (7) is reduced to

(12) $$\begin{array}{ll} \xi [M{]}^{2} & =2E\{(y_{R}-{\hat{x}}_{R}{)}^{2}\}\\ & =\frac{2}{k+1}\sum_{i=0}^{k}\sum_{j=0}^{k}\int_{D_{i,R}}{\left(y_{R}-S_{i,R}\right)}^{2}\frac{1}{\sigma_{n}}\varphi (\frac{y_{R}-\alpha S_{j,R}}{\sigma_{n}})dy_{R} \end{array}$$

Letting $v=y_{R}-S_{i,R}$, so ${\tilde{D}}_{i,R}=D_{i,R}-S_{i,R}$, and letting $\lambda_{ji,R}=-S_{i,R}+\alpha S_{j,R}$, Equation (12) can be reduce to

(13) $$\xi {\left[M\right]}^{2}=\frac{2}{k+1}\sum_{i=0}^{k}\sum_{j=0}^{k}\int_{{\tilde{D}}_{i,R}}\frac{v^{2}}{\sigma_{n}}\varphi (\frac{v-\lambda_{ji,R}}{\sigma_{n}})dv$$

and after some manipulation, Equation (13) is reduced to

(14) $$\begin{array}{ll} \xi [k{]}^{2}= & 2/(k+1)\{\\ & \sum_{j=0}^{k}\left[\sigma_{n}(-b+\lambda_{jk,R}\right)\varphi (\frac{-b-\lambda_{jk,R}}{\sigma_{n}})+(\lambda_{jk,R}^{2}+\sigma_{n}^{2})Q(\frac{-b-\lambda_{jk,R}}{\sigma_{n}})]\\ & +\sum_{j=0}^{k}\left[\sigma_{n}(b+\lambda_{jk,R}\right)\varphi (\frac{b-\lambda_{jk,R}}{\sigma_{n}})+(\lambda_{jk,R}^{2}+\sigma_{n}^{2})Q(\frac{b-\lambda_{jk,R}}{\sigma_{n}})]\\ & +\sum_{i=1}^{k}\sum_{j=0}^{k}\left[\sigma_{n}(-b+\lambda_{ji,R}\right)\varphi (\frac{-b-\lambda_{ji,R}}{\sigma_{n}})+(\lambda_{ji,R}^{2}+\sigma_{n}^{2})Q(\frac{-b-\lambda_{ji,R}}{\sigma_{n}})]\\ & -\sum_{i=1}^{k}\sum_{j=0}^{k}\left[\sigma_{n}(b+\lambda_{ji,R}\right)\varphi (\frac{b-\lambda_{ji,R}}{\sigma_{n}})+(\lambda_{ji,R}^{2}+\sigma_{n}^{2})Q(\frac{b-\lambda_{ji,R}}{\sigma_{n}})]\} \end{array}$$

where α is the attenuation factor with a PDF of $f_{\alpha ,\eta -\mu }$, and Q(⋅) is a complementary cumulative distribution function (CCDF). 

From Equation (14), the receiver can analyze the NDA-EVM value of different constellation size without requiring the transmitter to explicitly send each modulation order. In other words, if the receiver gets the NDA-EVM value of symbols with current modulation order, the NDA-EVM value for any other modulation order can be derived easily in the same channel condition (e.g., if the NDA-EVM for 16QAM is confirmed, the NDA-EVM for QAM/64QAM/256QAM can be obtained by using Equation (14) without retransmission at the same channel condition).

The key of adaptive modulation is to choose the optimum modulation order to match the channel conditions, while achieving the expected SER. To obtain the modulation order for which NDA-EVM can tolerate the expected SER, we need to confirm the relationship between NDA-EVM and SER. The SER as a function of NDA-EVM is derived in Appendix A, which is

(15) $$P_{S}=1-\{\frac{\xi {\left[k\right]}^{2}-F}{2\sigma_{n}^{2}}+\frac{1}{1+k}\sum_{i=1}^{k}\left[Q(\frac{-b-\lambda_{ii}}{\sigma_{n}})-Q(\frac{b-\lambda_{ii}}{\sigma_{n}})\right]{\}}^{2},$$

where

(16) $$\begin{array}{ll} F= & \frac{2}{1+k}\{\sum_{j=0}^{k}\left[\sigma_{n}(-b+\lambda_{jk,R})\varphi (\frac{-b-\lambda_{jk,R}}{\sigma_{n}})+\lambda_{jk,R}^{2}Q(\frac{-b-\lambda_{jk,R}}{\sigma_{n}})\right]+\sum_{j=0}^{k-1}\sigma_{n}^{2}Q(\frac{-b-\lambda_{jk,R}}{\sigma_{n}})\\ & +\sum_{j=0}^{k}\left[\sigma_{n}(b+\lambda_{jk,R})\varphi (\frac{b-\lambda_{jk,R}}{\sigma_{n}})+\lambda_{jk,R}^{2}Q(\frac{b-\lambda_{jk,R}}{\sigma_{n}})\right]+\sum_{j=0}^{k-1}\sigma_{n}^{2}Q(\frac{b-\lambda_{jk,R}}{\sigma_{n}})\\ & +\sum_{i=1}^{k}\sum_{j=0}^{k}\left[\sigma_{n}(-b+\lambda_{ji,R}\right)\varphi (\frac{-b-\lambda_{ji,R}}{\sigma_{n}})+(\lambda_{ji,R}^{2}+\sigma_{n}^{2})Q(\frac{-b-\lambda_{ji,R}}{\sigma_{n}})]\\ & -\sum_{i=1}^{k}\sum_{j=0}^{k}\left[\sigma_{n}(b+\lambda_{ji,R}\right)\varphi (\frac{b-\lambda_{ji,R}}{\sigma_{n}})+(\lambda_{ji,R}^{2}+\sigma_{n}^{2})Q(\frac{b-\lambda_{ji,R}}{\sigma_{n}})]\} \end{array}.$$

## 4. Adaptation Strategy Based on NDA-EVM

**Figure 2:**
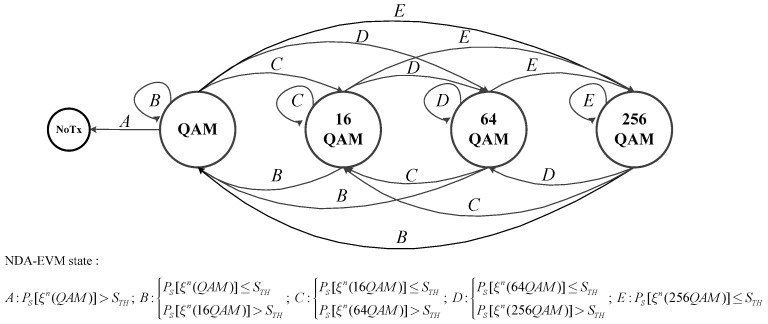
The state machine for NDA-EVM-AM.

Based on the previous NDA-EVM analysis, an adaptation strategy is proposed as follows. The NDA-EVM-AM for WSN is modeled as a state machine where each state is represented by a couple formed by a modulation order. To determine the optimum modulation order $M^{n+1}$ to be used at time n+1, the NDA-EVM of all modulation orders MQAM signals at time n (i.e., $NDA-EVM_{all}=\{\xi^{n}(k)|k=(1,3,7,15)\}$) are calculated and their corresponding SER $P_{S}$ are compared with the thresholds $S_{TH}$. The state machine in Figure 2 illustrates NDA-EVM-AM of the five states represented, by the letters A, B, C, D and E. A is the worst state that cannot establish communication under target SER (e.g., NoTx, No Transmission). States B, C, D and E denote the states from the former stage to QAM, 16QAM, 64QAM and 256QAM, respectively.

The pseudocode for adaptation strategy based on NDA-EVM is shown in Algorithm 1. Note that, if transmitting in particularly bad fading channel states, even the modulated QAM symbols with the minimum constellation size will not satisfy the expected SER (i.e., $P_{S}(\xi^{n}[k(1)])>S_{TH}$), and the transmitted sensor node will not send any data (NoTx, No Transmission). In this case, the transmitted sensor node will remain the outage state at time n+1, until the new turn (i.e., time n+2) of adaptive adjustment.
**Algorithm 1:** The Pseudocode for NDA-EVM-AM Strategy.
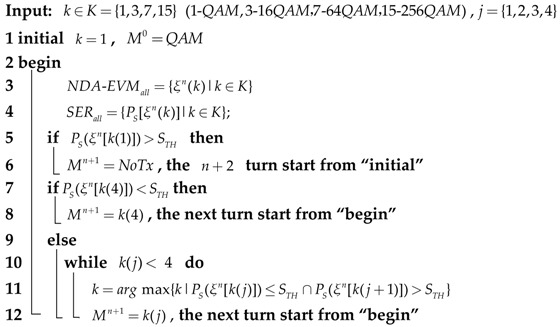


## 5. Algorithmic Complexity

Table 1 compares the computational complexity in each step of NDA-EVM-AM and DA-SNR-AM algorithm (proposed by [14]). Note that $M_{m}=\max(M)$ is the highest modulation order of MQAM signals, n is the number of modulation modes, and $n<M_{m}$.

Analyzing each step of NDA-EVM-AM algorithm, the processes with the highest complexity are calculation of NDA-EVM and SER with all constellation sizes, (i.e., both complexities are $ο(M_{m})$). Thus, NDA-EVM-AM algorithm has a complexity of $ο(M_{m})$, which is the same as the DA-SNR-AM algorithm.

Remarkably, in the aspect of constellation size adjustment, NDA-EVM-AM algorithm guarantee the transmitter can “jump” to the optimal constellation size “in one step”, thus has ο(1) operations. However, to optimize the set of switching levels, SNR-AM algorithm requires continuously refreshing the relationship of SNR-SER for every modulation mode, which requires ο(n) operations. Thus, the NDA-EVM-AM has less computational complexity in adaptation strategy, which means that NDA-EVM-AM has greater real-time in adjustment.

On the other hand, in the aspect of initializing the adaptive modulation system, the DA-SNR-AM algorithm needs to train known sequences (i.e., data aided symbols) to each modulation mode when the channel varies, which requires ο(n) operations, much more complex than the initialization of NDA-EVM-AM with ο(1), and the non-real time in train sequences will lose the accuracy of adaptive modulation. 

Above all, to improve the real-time and accuracy of adaptive modulation, NDA-EVM-AM algorithm is significantly complex in calculation of NDA-EVM values and SER with all constellation sizes. However, the computational complexity of NDA-EVM-AM is linear, and has high efficiency, even over rapidly time-varying channels.

## 6. Outage Capacity of NDA-EVM-AM in NLOS Scenario

The outage capacity is the realistic maximum of capacity of NDA-EVM-AM for WSN. It aims to achieve a maximum rate at which data can be transmitted over a fading channel with a certain outage probability $P_{out}$ [29]. Because the NLOS for WSN can be modeled by η−μ fading channels, for any 0≤ε<1, the outage capacity of NDA-EVM-AM of η−μ fading channels can be expressed as [23]:

(17) $$\begin{array}{l} C=\max\{R=\log_{2}(M_{\max}):P_{out}(R)\leq \varepsilon \},\\ M_{\max}=\xi_{\eta -\mu ,\min}^{-1}[M],\;M=(k+1{)}^{2} \end{array}.$$

This reflects the best-case performance of a realistic system in NLOS scenario of WSN. The outage capacity of NDA-EVM-AM calls for the use of the highest modulation order that can tolerate the lowest EVM on the η−μ fading channels at the receiver (i.e., the lower bound on the NDA-EVM) while achieving the expected SER such that the $P_{out}$ limit is satisfied.

NDA-EVM of the η−μ fading channels is given by

(18) $$\xi_{\eta -\mu }^{}(k)=\int_{0}^{1}\xi [M]f_{\alpha ,\eta -\mu }(\alpha )d\alpha ,$$

which is the η−μ fading channel’s NDA-EVM scaled by the specific probability of the occurrence of each instantaneous α, and averaged by integrating over the range of possible α. To obtain the lower bound of the NDA-EVM on the η−μ fading channels, we can lower bound ξ[k] by simplifying Equation (14) (as shown in the Appendix B), and it can be shown that

(19) $$\xi [k]>\frac{\sigma_{n}+bk}{{\left(2\pi \right)}^{1/4}}\exp(-\frac{b^{2}[{\left(1+k\right)}^{2}+2k(1+k)]+b^{2}k^{2}\alpha^{2}}{4\sigma_{n}^{2}}).$$

Substituting Equations (4) and (19) into Equation (18), the lower bound of NDA-EVM for a η−μ fading channel is (the derivation is shown in the Appendix C):

(20) $$\xi_{\eta -\mu }^{}(k)>\frac{1}{k^{2p}}\tau (k)\beta {\left(k\right)}^{2\mu -p}\frac{\Gamma (2\mu -p)}{\theta_{1}^{\mu }\theta_{2}^{\mu }\Gamma (2\mu )}F_{D}^{\left(2\right)}(2\mu -p,\mu ,\mu ;2\mu ;\frac{-\beta (k)}{\theta_{1}},\frac{-\beta (k)}{\theta_{2}}),k=\sqrt{M}-1.$$

In Equation (20), by setting the optimal $p=p^{*}$, we obtain

(21) $$p^{*}=\arg\underset{\begin{array}{l} p^{*}\in p\\ p\in (0,2\mu ) \end{array}}{\max}\{\beta {\left(k\right)}^{2\mu -p}\frac{\Gamma (2\mu -p)}{\theta_{1}^{\mu }\theta_{2}^{\mu }\Gamma (2\mu )}F_{D}^{\left(2\right)}(2\mu -p,\mu ,\mu ;2\mu ;\frac{-\beta (k)}{\theta_{1}},\frac{-\beta (k)}{\theta_{2}})\},$$

where p∈(0,2μ). 

After obtaining the lower bound of the NDA-EVM, by using the relationship between NDA-EVM and SER, the minimum NDA-EVM for a η−μ fading channel $\xi_{\eta -\mu ,\min}^{}(k)$ and the maximum modulation order $M_{\max}$ can be confirmed while satisfying the expected outage probability. Using the equations above and Equation (17), the outage capacity of an NLOS scenario for WSN is obtained. 

## 7. Performance Evaluation of NDA-EVM-AM on an NLOS Scenario 

Evaluating the performance of the adaptive modulation over a η−μ fading channel can provide a useful reference for the transmit design of WSN. The accuracy of evaluating the quality of a fading channel, the effectiveness of an adaptation strategy and the average spectral efficiency are the three most important performance indexes in an adaptive modulation system of WSN. To justify NDA-EVM-AM in an NLOS scenario, we employed the Monte Carlo approach to simulate MQAM signals transmitted over η−μ channels. We simulate the adaptive modulation with MQAM signals in physical uplink shared channel (PUSCH) specified in the 3GPP LTE-Hi standard. The simulation parameters are set as shown in Table 2.

### 7.1. Accuracy of Channel Quality Metric 

We consider the root of mean-square errors (RMSEs) as a standard of measuring the accuracy of channel quality metric. We compare the estimation of NDA-EVM and traditional metrics DA-SNR (e.g., there is one reference signal (RS) for every seven data symbols in PUSCH for a normal cyclic prefix (CP) over the η−μ channels). We transmit data over the η−μ fading channels with different channel parameter μ and η. For every η−μ fading channel, the estimated NDA-EVM (i.e., $\xi_{E}$) can be obtained using Equation (14). Meanwhile, we can measure the NDA-EVM (i.e., $\xi_{M}$) by the received symbols in the simulation as a real NDA-EVM (i.e., $\xi_{R}$), which is employed by Monte Carlo approach (i.e., n=1000 rounds in our simulation). The RMSE of NDA-EVM can be obtained as $RMSE=\sqrt{\sum {\left(\xi_{R}-\xi_{E}\right)}^{2}/n}$. In a similar way, the RMSE of DA-SNR can also be obtained.

**Figure 3:**
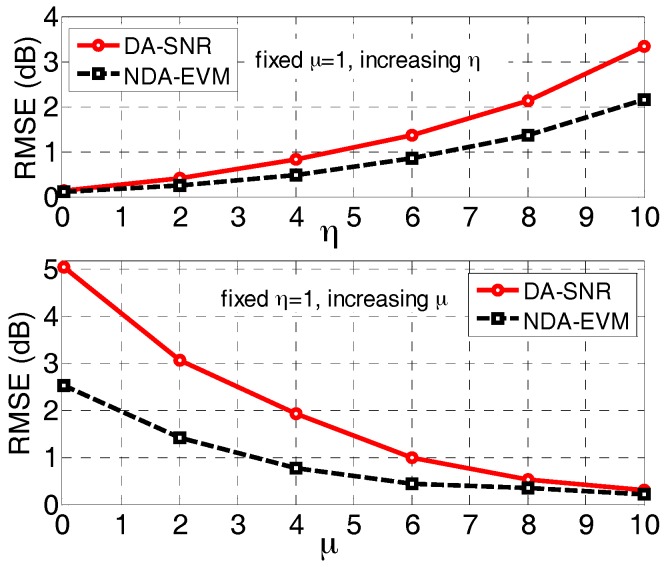
The root of mean-square error (RMSE) of channel quality metric estimation over η−μ fading channel.

The channel change frequency is a decreasing function of the channel parameter μ [30]. This causes the RMSE to increase with decreasing μ, as shown in Figure 3. The data-aided estimation accuracy is more sensitive to variations in μ because of the fixed time interval between RSs; by contrast, the NDA-EVM is more robust and has lower RMSEs at smaller μ values. Interestingly, η has the opposite effect on NDA-EVM as that of μ. Accordingly, the NDA-EVM estimation yields lower RMSEs compared with the DA-SNR estimation as the parameter η increases. As seen in Figure 3, the NDA-EVM is an effective metric for evaluating the quality of wireless fading channels in NLOS scenario.

### 7.2. Evaluation on Adaptation Strategy

We evaluated the accuracy of modulation order selection by NDA-EVM-AM and DA-SNR-AM in different NLOS scenarios of WSN. We transmitted data with every modulation order over every different η−μ fading channel. Under the target SER, the highest modulation order (i.e., $M_{Th}$) can be obtained in every η−μ fading channel. Executing NDA-EVM-AM and DA-SNR-AM at the same channel condition, the optimal modulation order for each scheme can be determined (i.e., $M_{N}$ is for NDA-EVM-AM, $M_{D}$ is for DA-SNR-AM). Comparing $M_{Th}$ and $M_{N}$/$M_{D}$, the accuracy of modulation order selection of NDA-EVM-AM/DA-SNR-AM is obtained.

**Figure 4:**
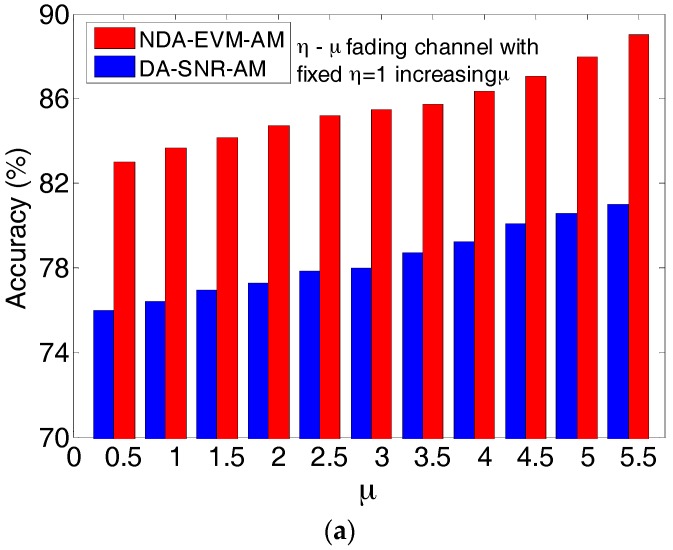

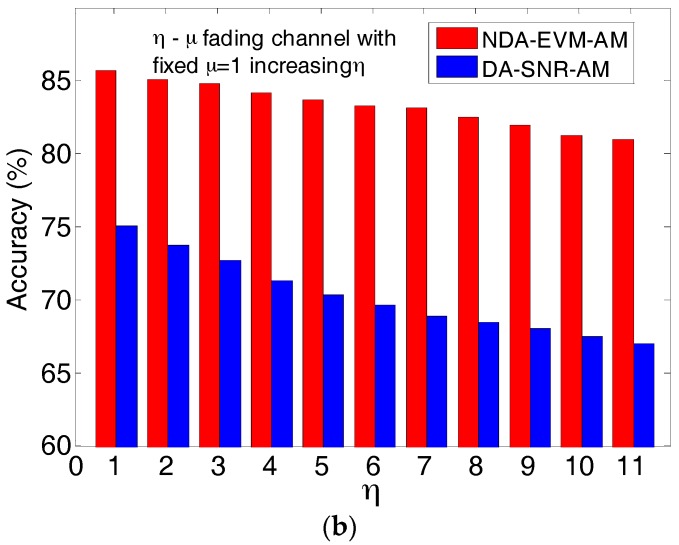
(**a**) The accuracy of modulation order selection for η−μ channels with fixed η=1 and increasing μ. (**b**) The accuracy of modulation order selection for η−μ channels with fixed μ=1 and increasing η.

Figure 4a,b shows the results in different η−μ channels with fixed η=1 and increasing μ, and fixed μ=1 and increasing η. Figure 4 illustrates that the accuracy of modulation order selection for NDA-EVM-AM is higher than that of DA-SNR-AM. This is because SNR is calculated only during the RS, which does not capture the entire packet duration. The relationship of SNR-SER cannot provide an accurate guidance for the modulation order selection. On the other hand, the channel changes more and more rapidly with the decreasing μ or increasing η. As a symbol-level performance metric, NDA-EVM has a real-time update for modulation order selection, which is different from DA-SNR. Moreover, the adaptation strategy requires a modulation order selection scheme to jump multiple levels in one step under the rapid-changing channel; NDA-EVM-AM executes these jumps more effectively, which can be observed in Section 4.

**Figure 5:**
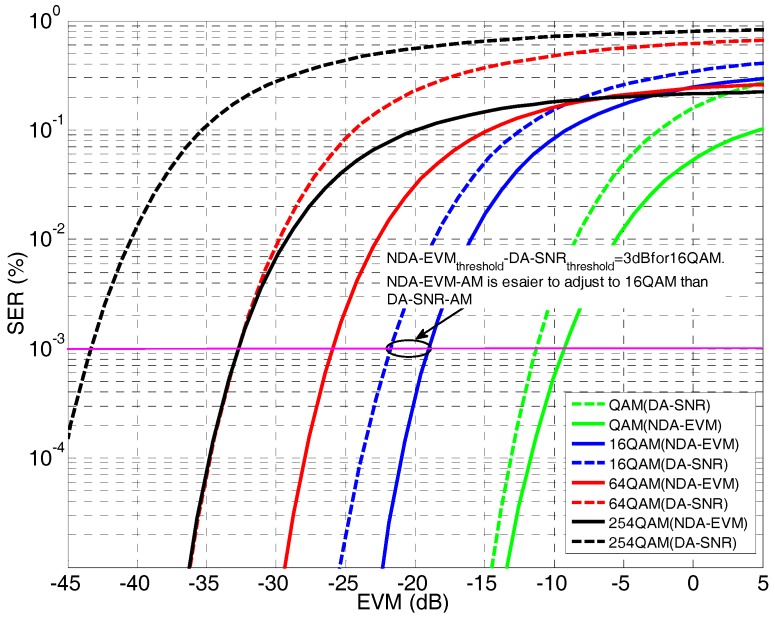
The comparison of switching thresholds for the two different adaptation strategies based on SER curves.

We compare the switching thresholds for the two different adaptation strategies for the NLOS scenarios of WSN. In Figure 5, both SER-NDA-EVM and SER-DA-SNR (which can be translated into the EVM using the formula $EVM=\sqrt{1/SNR}$ [31]) are presented under the η−μ channel with parameter values of η=1 and μ=0.5 (i.e., Rayleigh fading channel). It can be seen that, for the target SER, the switching threshold based on the NDA-EVM is easier to achieve than that of DA-SNR at the same modulation order. For example, the 16QAM switching threshold in terms of the NDA-EVM is −19 dB, whereas, for the DA-SNR, it is −22 dB, which implies that NDA-EVM-AM is easier to adjust to 16QAM. In other words, NDA-EVM-AM can be used to transmit 16QAM signals under the target SER, whereas DA-SNR-AM must remain at the next lower modulation order even under the same channel conditions. This effect illustrates that NDA-EVM-AM has a higher average spectral efficiency than that of DA-SNR-AM in NLOS scenarios.

### 7.3. Average Spectral Efficiency 

**Figure 6:**
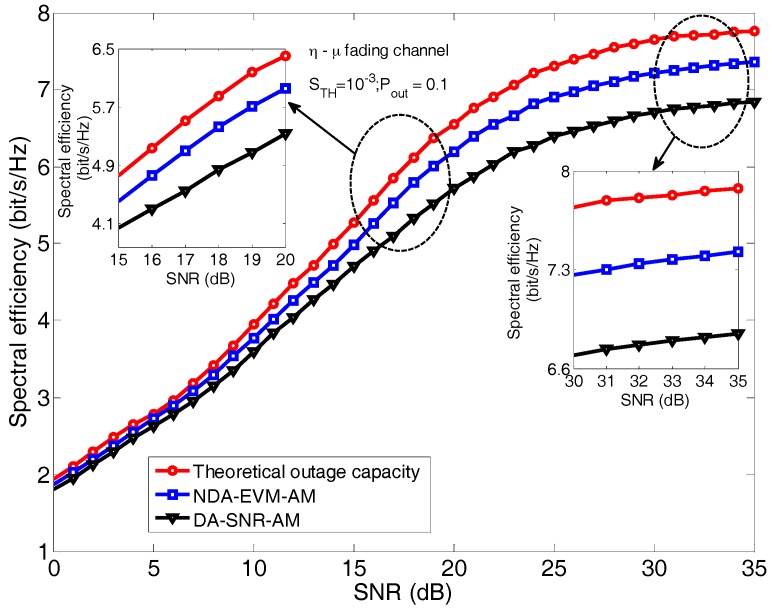
The average spectral efficiency comparison for NDA-EVM-AM and DA-SNR-AM.

The average spectral efficiency of two adaptive modulation schemes at target $P_{out}=0.1$, $S_{TH}=10^{-3}$ in NLOS scenario (i.e., the η−μ channel with parameter values of η=1 and μ=0.5), along with the theoretical outage capacity, is shown in Figure 6. Specifically, the theoretical outage capacity is the upper bound of the average spectral efficiency of adaptive modulation, which can be obtained by using the method in Section 5. In Figure 6, it is shown that NDA-EVM-AM is closer to the theoretical outage capacity than DA-SNR-AM. Although both schemes suffer performance degradation within the low SNR region, NDA-EVM-AM has a 0.4 bit/s/Hz increment of spectral efficiency over DA-SNR-AM in the middle SNR region and a 0.8 bit/s/Hz increment in the high SNR region. From the analysis in the Section 7.2, NDA-EVM-AM has a more accurate modulation order selection over DA-SNR-AM, while the switching thresholds of NDA-EVM-AM lead to a more efficient modulation order adjustment than that of DA-SNR-AM. Overall, NDA-EVM-AM is an effective adaptive modulation for an NLOS scenario of WSN. 

## 8. Conclusions

In this paper, NDA-EVM-AM is proposed for the adaptive modulation in an NLOS scenario of WSN. We consider NDA-EVM as a new performance metric for evaluating fading channels, and model the NLOS scenario as the η−μ fading channel. A closed-form expression for the NDA-EVM of MQAM signal over η−μ fading channels is derived, and the relationship between NDA-EVM and SER is also formulated. Based on the NDA-EVM performance in the NLOS scenario, a NDA-EVM state machine is designed for adaptation strategy. The algorithmic complexity of NDA-EVM-AM is analyzed and the outage capacity of NDA-EVM-AM in an NLOS scenario is also given. Finally, the performances of NDA-EVM-AM are compared by simulation, and the results show that NDA-EVM is an effective metric to quantify an NLOS link, while the adaptation strategy based on the NDA-EVM state machine is proven more efficient than traditional adaptive modulation. These effects contribute to a higher average spectral efficiency in an NLOS scenario of WSN.

## Figures and Tables

**Table 1:** Comparison of algorithmic complexity.

Steps	NDA-EVM-AM Complexity	DA-SNR-AM Complexity
Initialization	ο(1)	ο(n)
Calculation of the metric	$ο(M_{m})$	ο(n)
Calculation of SER	$ο(M_{m})$	$ο(M_{m})$
Adjustment of constellation size	ο(1)	ο(n)
Complexity of algorithm	$ο(M_{m})$	$ο(M_{m})$

**Table 2:** Simulation parameters of wireless transmitter

Parameters	Value
Target SER	0.001
Outage probability $P_{out}$	0.1
Radio frequency carrier	2.4 GHz
Transmitter power $P_{0}$	1 W
Symbol period $T_{sym}$	66.7 µs
Data-aided interval $T_{DA}$	0.5 ms
Constellation size of MQAM	QAM/16QAM/64QAM/256QAM

## Appendix A

Considering the independence and symmetry between the real and imaginary parts of the MQAM signal, the probability of the symbol being correctly received for the in-phase and quadrature branches of the MQAM signal are equal, $P_{C,R}=P_{C,I}$. Therefore, the SER for MQAM signal is

(A1) $$P_{S}=1-P_{C,R}^{2}$$

Because the symbol estimator tends to assign received symbols to their closest possible constellation point, the probability of the transmitted symbol $S_{i}$, which is correctly estimated as $\hat{x}=S_{i}$ by the receiver, can be expressed as (only the real part is considered)

(A2) $$\begin{array}{l} P_{C,R}=\frac{1}{k+1}\sum_{i=0}^{k}P_{C,R}(S_{i,R})\\ =\frac{1}{k+1}\sum_{i=0}^{k}P({\hat{x}}_{R}=S_{i,R}|x_{R}=S_{i,R})\\ =\frac{1}{k+1}\sum_{i=0}^{k}\int_{D_{i,R}}f(y_{R}|x_{R}=S_{i,R})dy_{R}=\frac{1}{k+1}\sum_{i=0}^{k}\int_{D_{i,R}}\frac{1}{\sigma_{n}}\varphi (\frac{y_{R}-\alpha S_{i,R}}{\sigma_{n}})dy_{R} \end{array},$$

where $D_{i,R}$ is the decision region for symbol $S_{i,R}$. By using $v=y_{R}-S_{i,R}$, then ${\tilde{D}}_{iR}=D_{iR}-S_{iR}$, $y_{R}-\alpha S_{iR}=v-(-S_{iR}+\alpha S_{iR})$. Letting $\lambda_{ii,R}=-S_{i,R}+\alpha S_{i,R}$, Equation (A2) can be rewritten as

(A3) $$\begin{array}{l} P_{C,R}=\frac{1}{k+1}\sum_{i=0}^{k}\int_{{\tilde{D}}_{iR}}\frac{1}{\sigma_{n}}\phi (\frac{v-\lambda_{ii}}{\sigma_{n}})dv\\ =\frac{1}{k+1}Q(\frac{-b-\lambda_{kk}}{\sigma_{n}})+\frac{1}{k+1}Q(\frac{b-\lambda_{kk}}{\sigma_{n}})+\frac{1}{k+1}\sum_{i=1}^{k}(Q(\frac{-b-\lambda_{ii}}{\sigma_{n}})-Q(\frac{b-\lambda_{ii}}{\sigma_{n}})) \end{array}.$$

Comparing Equations (14) and (A3), we can get

(A4) $$P_{C,R}=\frac{\xi {\left[k\right]}^{2}-F}{2\sigma_{n}^{2}}+\frac{1}{k+1}\sum_{i=1}^{k}(Q(\frac{-b-\lambda_{ii}}{\sigma_{n}})-Q(\frac{b-\lambda_{ii}}{\sigma_{n}})),$$

where

(A5) $$\begin{array}{ll} F= & \frac{2}{1+k}\{\sum_{j=0}^{k}\left[\sigma_{n}(-b+\lambda_{jk,R})\varphi (\frac{-b-\lambda_{jk,R}}{\sigma_{n}})+\lambda_{jk,R}^{2}Q(\frac{-b-\lambda_{jk,R}}{\sigma_{n}})\right]+\sum_{j=0}^{k-1}\sigma_{n}^{2}Q(\frac{-b-\lambda_{jk,R}}{\sigma_{n}})\\ & +\sum_{j=0}^{k}\left[\sigma_{n}(b+\lambda_{jk,R})\varphi (\frac{b-\lambda_{jk,R}}{\sigma_{n}})+\lambda_{jk,R}^{2}Q(\frac{b-\lambda_{jk,R}}{\sigma_{n}})\right]+\sum_{j=0}^{k-1}\sigma_{n}^{2}Q(\frac{b-\lambda_{jk,R}}{\sigma_{n}})\\ & +\sum_{i=1}^{k}\sum_{j=0}^{k}\left[\sigma_{n}(-b+\lambda_{ji,R}\right)\varphi (\frac{-b-\lambda_{ji,R}}{\sigma_{n}})+(\lambda_{ji,R}^{2}+\sigma_{n}^{2})Q(\frac{-b-\lambda_{ji,R}}{\sigma_{n}})]\\ & -\sum_{i=1}^{k}\sum_{j=0}^{k}\left[\sigma_{n}(b+\lambda_{ji,R}\right)\varphi (\frac{b-\lambda_{ji,R}}{\sigma_{n}})+(\lambda_{ji,R}^{2}+\sigma_{n}^{2})Q(\frac{b-\lambda_{ji,R}}{\sigma_{n}})]\} \end{array}$$

Substituting Equation (A4) into Equation (A1), the SER as a function of NDA-EVM is

(A6) $$P_{S}=1-\{\frac{\xi [k{]}^{2}-F}{2\sigma_{n}^{2}}+\frac{1}{1+k}\sum_{i=1}^{k}\left[Q(\frac{-b-\lambda_{ii}}{\sigma_{n}})-Q(\frac{b-\lambda_{ii}}{\sigma_{n}})\right]{\}}^{2}.$$

## Appendix B

The expression for the NDA-EVM given in Equation (14) can be viewed as the sum of two error sums. The first sum consists of a sum of the errors over singly truncated decision regions (i.e., $D_{0,R}$ and $D_{k,R}$), and the second sum consists of a sum of the errors over doubly truncated decision regions $D_{i,R}$. Because the second sum is always positive and its value is much smaller than that of the first sum, Equation (14) can be reduced to

(A7) $$\begin{array}{ll} \xi [k{]}^{2}>\frac{2}{k+1}\{\sum_{j=0}^{k} & \left[\sigma_{n}(-b+\lambda_{jk,R}\right)\varphi (\frac{-b-\lambda_{jk,R}}{\sigma_{n}})+(\lambda_{jk,R}^{2}+\sigma_{n}^{2})Q(\frac{-b-\lambda_{jk,R}}{\sigma_{n}})]\\ & +\sum_{j=0}^{k}\left[\sigma_{n}(b+\lambda_{jk,R}\right)\varphi (\frac{b-\lambda_{jk,R}}{\sigma_{n}})+(\lambda_{jk,R}^{2}+\sigma_{n}^{2})Q(\frac{b-\lambda_{jk,R}}{\sigma_{n}})]\} \end{array}.$$

Considering $\lambda_{jk,R}=b[\alpha (j-k)+(\alpha j-k)]<0$ and Q(x) decrease monotonically. Therefore, $Q[(-b-\lambda_{jk,R})/\sigma_{n}]>Q[(b-\lambda_{jk,R})/\sigma_{n}]$ similarly, $\varphi [(-b-\lambda_{jk,R})/\sigma_{n}]>\varphi [(b-\lambda_{jk,R})/\sigma_{n}]$.

From the above and by using φ(x)≤Q(x) after manipulation, Equation (A7) becomes

(A8) $$\xi {\left[M\right]}^{2}>\frac{1}{k+1}\sum_{j=0}^{k}\frac{(\sigma_{n}-\lambda_{jk,R}{)}^{2}}{\sqrt{2\pi }}\exp(-(\frac{b-\lambda_{jk,R}}{\sqrt{2}\sigma_{n}}{)}^{2}).$$

Considering that ${\left(1+k-(2j-k)\alpha \right)}_{\max}=1+k\alpha +k$, Equation (A8) can be rewritten as

(A9) $$\xi {\left[k\right]}^{2}>\frac{1}{k+1}\sum_{j=0}^{k}\frac{{\left(\sigma_{n}-\lambda_{jk,R}\right)}^{2}}{\sqrt{2\pi }}\exp(-\frac{b^{2}k^{2}\alpha^{2}}{2\sigma_{n}^{2}})\exp(-\frac{b^{2}[{\left(1+k\right)}^{2}+2k(1+k)]}{2\sigma_{n}^{2}}).$$

According to the Cauchy–Schwarz–Buniakowsky inequality [32], $(\sum_{k=1}^{n}a_{k}b_{k}{)}^{2}\leq \sum_{k=1}^{n}a_{k}^{2}\sum_{k=1}^{n}b_{k}^{2}$. Let $b_{k}=1$, we get $(\sum_{k=1}^{n}a_{k}{)}^{2}\leq \;n\sum_{k=1}^{n}a_{k}^{2}$, and Equation (A9) can be reduced to

(A10) $$\xi [k]>\frac{1}{\left(k+1\right)}\exp(-\frac{b^{2}[{\left(1+k\right)}^{2}+2k(1+k)]}{4\sigma_{n}^{2}})\exp(-\frac{b^{2}k^{2}\alpha^{2}}{4\sigma_{n}^{2}})(\sum_{j=0}^{k}\frac{\left(\sigma_{n}+bk\right)}{{\left(2\pi \right)}^{1/4}}+\sum_{j=0}^{k}\frac{\left(-(2j-k)bk\right)}{{\left(2\pi \right)}^{1/4}})$$

Since $\sum_{j=0}^{k}(-(2j-k)bk)/{\left(2\pi \right)}^{1/4}=0$, we can obtain the lower bound on the NDA-EVM as follows:

(A11) $$\xi [M]>\frac{\sigma_{n}+bk}{{\left(2\pi \right)}^{1/4}}\exp(-\frac{b^{2}[{\left(1+k\right)}^{2}+2k(1+k)]+b^{2}k^{2}\alpha^{2}}{4\sigma_{n}^{2}}).$$

## Appendix C

The lower bounds of NDA-EVM over the η−μ channels can be obtained by substituting Equations (4) and (A11) into Equation (18), and letting $t=b^{2}k^{2}\alpha^{2}/4\sigma_{n}^{2}$, it can be rewritten as

(A12) $$\xi_{\eta -\mu }^{}(k)>\int_{0}^{\infty }\frac{\tau (k)e^{-t}}{\theta_{1}^{\mu }\theta_{2}^{\mu }\Gamma (2\mu )}\beta {\left(k\right)}^{2s}t^{2\mu -1}\Phi_{2}^{\left(2\right)}(\mu ,\mu ;2\mu ;\frac{-\beta (k)t}{\theta_{1}},\frac{-\beta (k)t}{\theta_{2}})dt,$$

where $\tau (k)=\frac{\sigma_{n}+bk}{{\left(2\pi \right)}^{1/4}}\exp(-\frac{b^{2}[{\left(1+k\right)}^{2}+2k(1+k)]}{4\sigma_{n}^{2}})$ and $\beta (k)=\frac{4\sigma_{n}^{2}}{b^{2}k^{2}}$. Without the loss of generality, p is introduced, and Equation (A11) is transformed as follows:

(A13) $$\xi_{\eta -\mu }^{}(k)>\int_{0}^{\infty }\frac{\tau (k)e^{-t}}{\theta_{1}^{\mu }\theta_{2}^{\mu }\Gamma (2\mu )}\beta {\left(k\right)}^{2\mu -p}\frac{1}{k^{2p}}{\left(\frac{2\sigma_{n}}{b}\right)}^{2p}t^{2\mu -1-p}t^{p}\Phi_{2}^{\left(2\right)}(\mu ,\mu ;2\mu ;\frac{-\beta (k)t}{\theta_{1}},\frac{-\beta (k)t}{\theta_{2}})dt.$$

When α≥1/k and $t^{p}{\left(2\sigma_{n}/b\right)}^{2p}={\left(k\alpha \right)}^{2p}\geq 1$, Equation (34) can be rewritten as:

(A14) $$\xi_{\eta -\mu }^{}(k)>\int_{0}^{\infty }\frac{1}{k^{2p}}\tau (k)\beta {\left(k\right)}^{2\mu -p}\exp(-t)\frac{t^{2\mu -p-1}}{\theta_{1}^{\mu }\theta_{2}^{\mu }\Gamma (2\mu )}\Phi_{2}^{\left(2\right)}(\mu ,\mu ;2\mu ;\frac{-\beta (k)t}{\theta_{1}},\frac{-\beta (k)t}{\theta_{2}})dt.$$

According to the identity [28]

(A15) $$F_{D}^{\left(n\right)}[a,b_{1},\cdot \cdot \cdot ,b_{n},c,x_{1},\cdot \cdot \cdot ,x_{n}]=\frac{1}{\Gamma (a)}\int_{t=0}^{\infty }e^{-t}t^{a-1}\Phi_{2}^{\left(n\right)}(b_{1},\cdot \cdot \cdot ,b_{n},c,x_{1}t,\cdot \cdot \cdot ,x_{n}t)dt,$$

Equation (A14) is equivalent to

(A16) $$\xi_{\eta -\mu }^{}(k)>\frac{1}{k^{2p}}\tau (k)\beta {\left(k\right)}^{2\mu -p}\frac{\Gamma (2\mu -p)}{\theta_{1}^{\mu }\theta_{2}^{\mu }\Gamma (2\mu )}F_{D}^{\left(2\right)}(2\mu -p,\mu ,\mu ;2\mu ;\frac{-\beta (k)}{\theta_{1}},\frac{-\beta (k)}{\theta_{2}}).$$

In Equation (A16), by setting the optimal $p=p^{*}$, we obtain

(A17) $$p^{*}=\arg\underset{\begin{array}{l} p^{*}\in p\\ p\in (0,2\mu ) \end{array}}{\max}\{\beta {\left(k\right)}^{2\mu -p}\frac{\Gamma (2\mu -p)}{\theta_{1}^{\mu }\theta_{2}^{\mu }\Gamma (2\mu )}F_{D}^{\left(2\right)}(2\mu -p,\mu ,\mu ;2\mu ;\frac{-\beta (k)}{\theta_{1}},\frac{-\beta (k)}{\theta_{2}})\},$$

where p∈(0,2μ).
